# Structural and electrical properties of oxygen complexes in Cz and FZ silicon crystals implanted with carbon ions

**DOI:** 10.1186/1556-276X-9-693

**Published:** 2014-12-23

**Authors:** Boris Romanyuk, Victor Melnik, Valentin Popov, Vilik Babich, Vasyl Kladko, Olexandr Gudymenko, Volodimir Ilchenko, Iegor Vasyliev, Andrii Goriachko

**Affiliations:** 1 V. Lashkarev Institute of Semiconductor Physics NAS of Ukraine, 41 Prospect Nauki, 03028 Kyiv, Ukraine; 2 Institute of High Technologies of Kyiv National T. Shevchenko University, 2 Glushkov av, 01601 Kyiv, Ukraine

**Keywords:** Silicon, Thermal donor, Ion implantation, Oxygen, Carbon, P-n junction

## Abstract

34.50.Dy; 61.10.-i; 68.35.Dv

**Electronic supplementary material:**

The online version of this article (doi:10.1186/1556-276X-9-693) contains supplementary material, which is available to authorized users.

## Background

Low-energy (1 to 20 keV) ion implantation of various impurities into Si is commonly used to make super-shallow *p-n* junctions[1, 2] and to form a variety of quantum-sized objects[3, 4]. Implantation of dopants such as B, P, As, and Sb into Si is usually made through a thin (3 to 5 nm) silicon oxide protective (screen) layer. Interaction of accelerated ions with oxygen atoms inside the screen oxide layer leads to generation of recoil oxygen atoms. The latter penetrate into Si substrate and stimulate the quasi-chemical reactions of matrix atoms and point defects in the Si surface layer. In particular, it was shown[5] that oxygen atoms influence the processes of As deactivation and its segregation at the SiO_2_-Si interface. The effect of recoil atoms is especially important when using ion implantation to form nanoscale objects in the surface region. Also, a significant number of carbon atoms are incorporated into a silicon matrix, because the absorption of carbon contaminants always occurs during the process of ion implantation.

It is known that the presence of substitution carbon atoms C_s_ impacts the oxygen precipitation and the associated defect formation processes[6, 7]. A small covalent radius *R*_c_ (0.077 nm) of C atoms (as compared to Si lattice atoms, *R*_c_ = 0.117 nm) causes a tensile strain within the Si matrix around C_s_ atoms and influences the process of TD formation[8].

A necessary condition for the TD center formation is the presence of certain oxygen amount in the carbon-implanted layer. It was shown in[9] that a high-energy implantation of carbon into *p*-type Cz-Si creates a layer of *n*-type conductivity in the region of carbon presence, while the implantation into the FZ-Si does not lead to such kind of doping. When carbon is implanted through a thin oxide layer, the incorporation of oxygen atoms into the Si matrix is taking place, due to recoil generation. Depending on the implantation dose, the concentration of these oxygen atoms in the Si surface layer (in the FZ-Si) can be > 10^18^ cm^-3^, which is comparable to the oxygen concentration in Cz-Si crystals. This can affect the generation of thermal donor centers, even in the FZ-Si samples. In this paper, we compare the TD center formation in silicon obtained by the float zone and Czochralski methods at low-energy ion implantation of carbon. The kinetics of the TD center formation and the structural transformation of the Si matrix during annealing have been investigated. We point out an important role of recoil oxygen atoms in the TD center formation in FZ-Si crystals.

## Methods

The Si(100) wafers of *p*-Cz-Si (10 Ohm × cm), *p*-FZ-Si (2 Ohm × cm), and *n*-FZ-Si (3,000 Ohm × cm) with the bulk oxygen concentration of 1 × 10^18^ (Si-Cz) and 5 × 10^16^ cm^-3^ (Si-FZ) were used in our experiments. After the standard surface treatment, they were oxidized at *T* = 800°C in dry O_2_ ambient in order to obtain the 5-nm oxide thickness. One portion of the wafers was etched in HF to remove the SiO_2_ layer. These, as well as the non-etched wafers, were placed into the vacuum chamber of the ion implanter. A sequential ion implantation of C^+^ ions with the energies 25, 50, and 90 keV and with doses in proportion of 2: 3: 6, respectively, was performed. The dose for each implantation energy was chosen to obtain a BOX profile with carbon concentration in the range from 2 × 10^17^ to 1 × 10^19^ cm^-3^. Some of the FZ-Si samples were additionally implanted with 130 keV$O_{2}^{+}$ ions to 1 × 10^18^ cm^-3^ concentration. After implantation, the surface oxide was removed followed by 30-min annealing in vacuum within the temperature range from 520°C to 800°C. To measure the current-voltage (*I-V*) and capacitance-voltage (*C-V*) characteristics, we have manufactured the test structures with an upper (field) Al electrode (Ø 3 mm). The *C-V* characteristics were measured using the Agilent Technology Analyzer 4294A (Agilent Technologies, Santa Clara, CA) at 500 kHz. Such a frequency is considered adequate to evade the impact of the surface electronic states on the capacitance charging/discharging processes. Thus, the appearance of additional peaks in the *C-V* characteristic may only be caused by the carrier exchange among the local states in the Si crystal. Special test structures with the surface oxide thickness of approximately 30 nm were manufactured to investigate the influence of temperature on the *C-V* parameters. The resulting doping level due to thermal donor center presence was determined from a surface resistance measured by the 4-probe method. The peculiarities of micro-defect creation in the implanted region of the Si bulk were studied by the diffuse X-ray scattering (DXS) technique, which provided the information about the defects' types, sizes, and mechanical stress fields.

## Results

**Figure 1:**
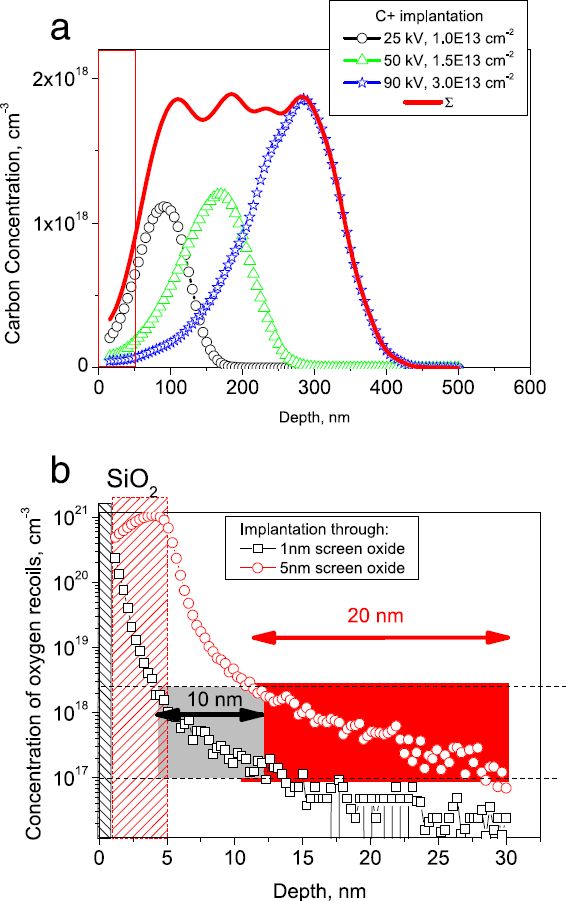
**Depth profiles of carbon implanted impurity (a) and oxygen recoil atoms (b).**

Figure 1a shows a TRIM 2008[10] calculated profile of carbon distribution in the silicon matrix for the implantation energies of 25, 50, and 90 keV and the total distribution of atoms after the multi-energy implantation. For given conditions, the average concentration of implanted carbon is close to 2 × 10^18^ cm^-3^. The distribution of recoil oxygen atoms for screen oxide thicknesses of 1 and 5 nm is shown in Figure 1b for the implantation dose of 5 × 10^14^ cm^-2^.

The dotted lines at 1 × 10^17^ and 2 × 10^18^ cm^-3^ oxygen concentrations encompass a region of optimum conditions for TD center generation. Outside of this region, the oxygen concentrations >2 × 10^18^ cm^-3^ lead to large SiO_2_ precipitates formation (due to oxygen supersaturation), while the concentrations <1 × 10^17^ cm^-3^ are insufficient for TD formation. After the implantation through the 5-nm screen oxide, the oxygen concentration is optimal in approximately 20-nm thick region. For 1-nm screen oxide, such region is only approximately 10-nm thick. Therefore, at low (25 keV) implantation energy, the distribution of carbon atoms is roughly the same as for the oxygen recoils, thus stimulating the TD creation.

**Figure 2:**
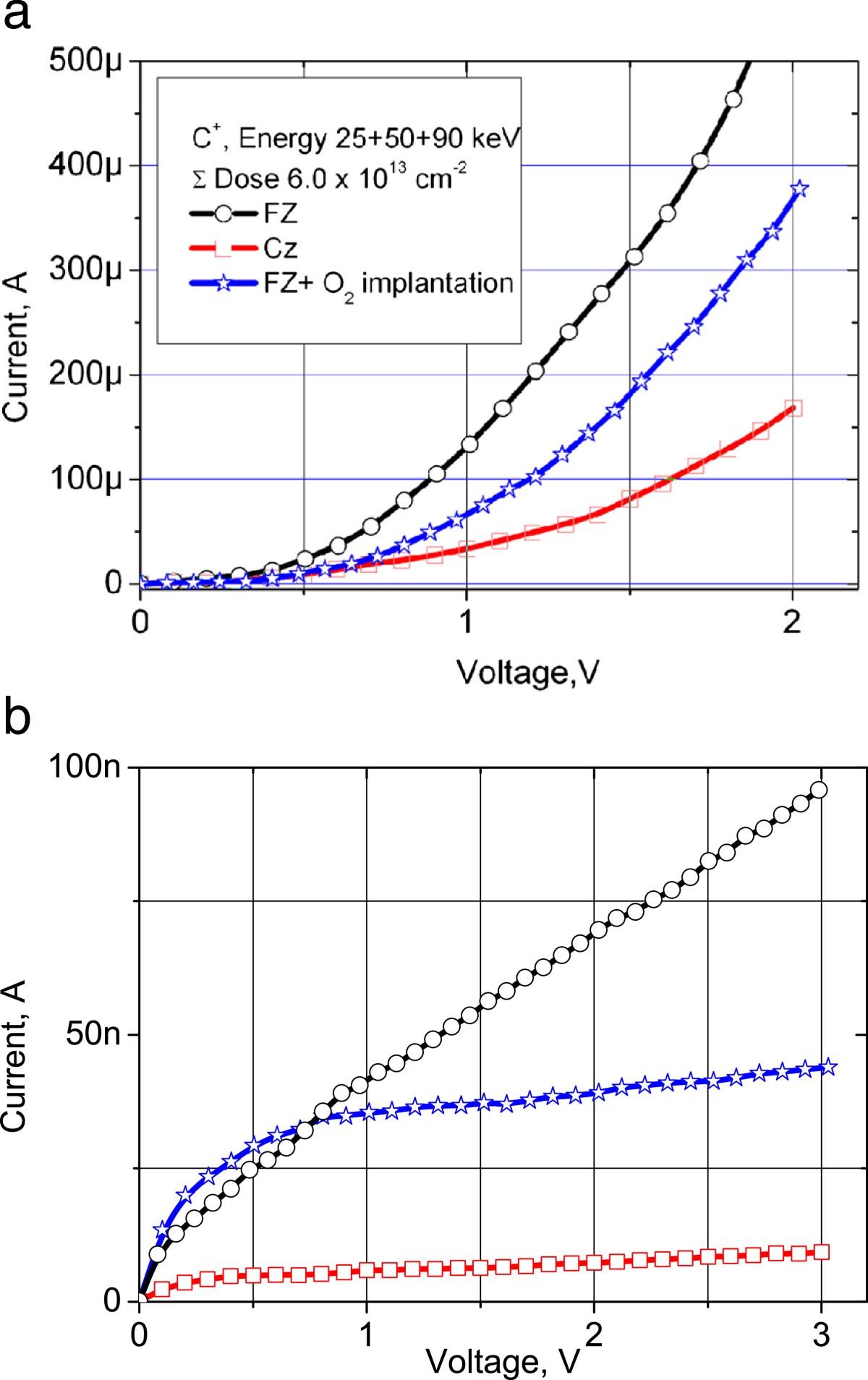
***I-V*** **characteristics of the fabricated diode structures.** Forward **(a)** and reverse **(b)** branches of the current-voltage characteristics for diode structures fabricated in Cz-Si silicon, FZ-Si, and FZ-Si additionally implanted by oxygen ions.

Figure 2 illustrates the *I-V* characteristics of the diode test structures fabricated in carbon-implanted Cz and FZ silicon as well as a separate FZ sample implanted both with carbon and oxygen ions. The forward branches of the *I-V* characteristics confirm the *p-n* junction presence, while the slope is the highest for the FZ-Si diode (Figure 2a). The structures fabricated on the basis of the Cz-Si demonstrate the smallest reverse current (approximately 10 nA @ 3 V) (Figure 2b) and the highest breakdown voltage of approximately 50 V. In contrast to this, the structures fabricated from the FZ-Si wafers deliver a high leakage current (approximately 100 nA @ 3 V) and an early breakdown at as low as 8-V reverse bias. The additional oxygen implantation into the FZ-Si decreases the leakage currents substantially and increases the breakdown voltages up to the value of 20 V.

**Figure 3:**
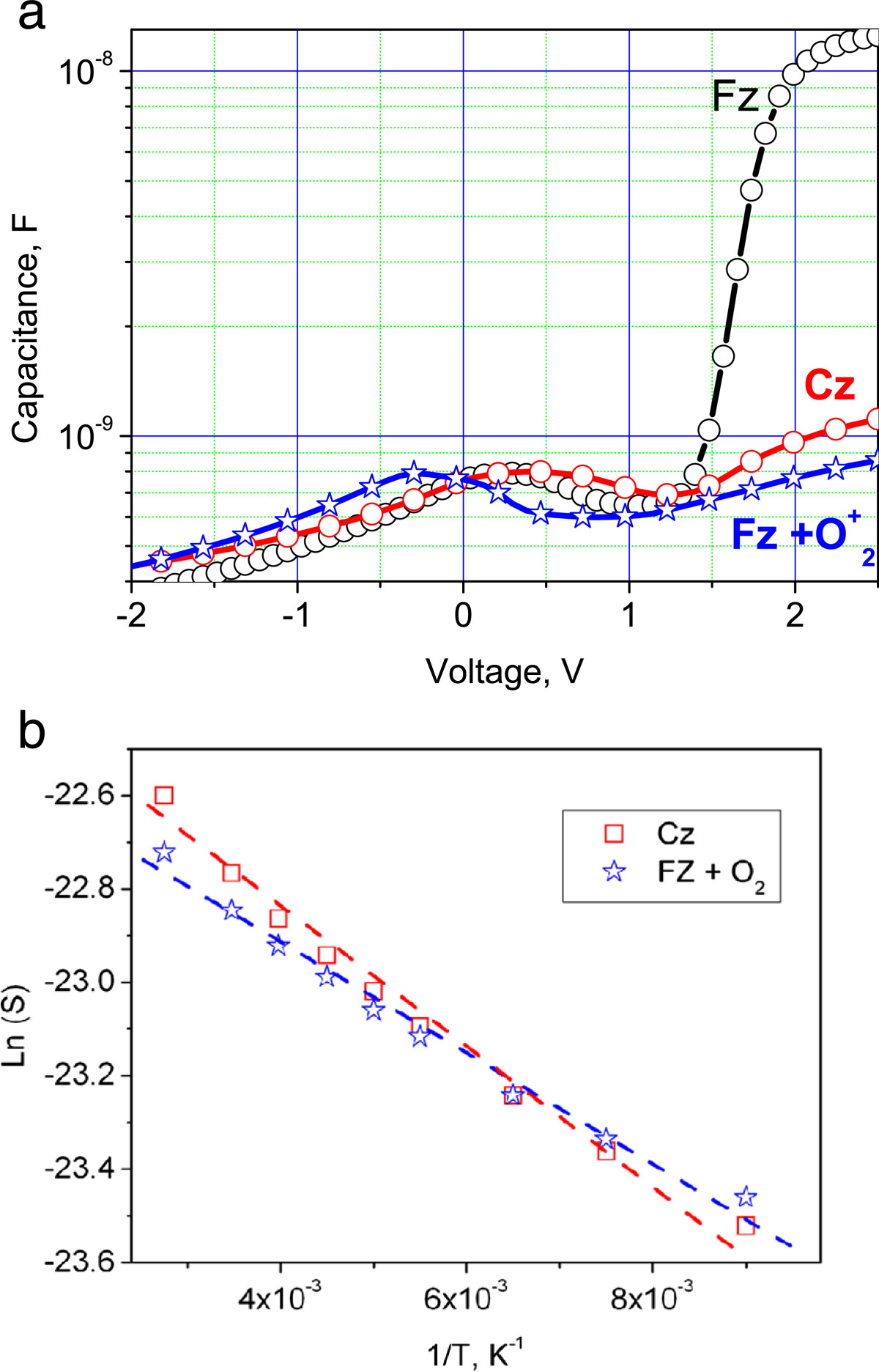
***C-V*** **characteristics of the fabricated diode structures.** The capacitance-voltage characteristics of the diode structures fabricated in the FZ-Si, Cz-Si, and FZ-Si additionally implanted with O_2_^+^ ions **(a)**. The dependence of capacity peak area changes as a function of inverse temperature **(b)**.

The *C-V* characteristics of the FZ-Si, Cz-Si, and FZ-Si + implanted oxygen cases are shown in Figure 3. At reverse voltages, the capacitance of all samples is typically decreased, due to the general contribution of the *p-n* junction's depletion region. One can observe a capacitance peak caused by recharging of TD states in the bias region from -0.5 to +0.5 V, for all samples. For direct bias, the capacitance increases due to the contribution from the forward-biased *p-n* junction. A particularly sharp increase in capacitance occurs in the structures fabricated on the FZ-Si wafer. After additional oxygen implantation into the FZ-Si-based structures, the shape of *C-V* characteristics is similar to those of the Cz-Si-based structures, except for a slight shift of the capacitance peak toward more negative bias value.

Using a numerical integration, we have obtained the area below the peak on the *С(V)* curve:$Q=\int_{V_{1}}^{V_{2}}C\left(V\right)\text{dV}$, where *V*_1_ and *V*_2_ are the interval boundaries containing the TD-related peak. From the physical point of view, the integral value corresponds to the electrical charge accumulated in the local TD states. Our numerical analysis has revealed an exponential dependence of this charge on the reciprocal temperature. To determine the energy position of states responsible for the peak's appearance on the *C-V* curve, we plot the dependence of electrical charge accumulated in the TD local states on inverse temperature. The Arrhenius plot, built according to the formula$\ln\;Q=f\left(\frac{1}{T}\right)$ between 100 and 300 K, yields an activation energy of 0.012 eV for Cz-Si and 0.014 eV for FZ-Si + implanted oxygen samples (Figure 3b).

**Figure 4:**
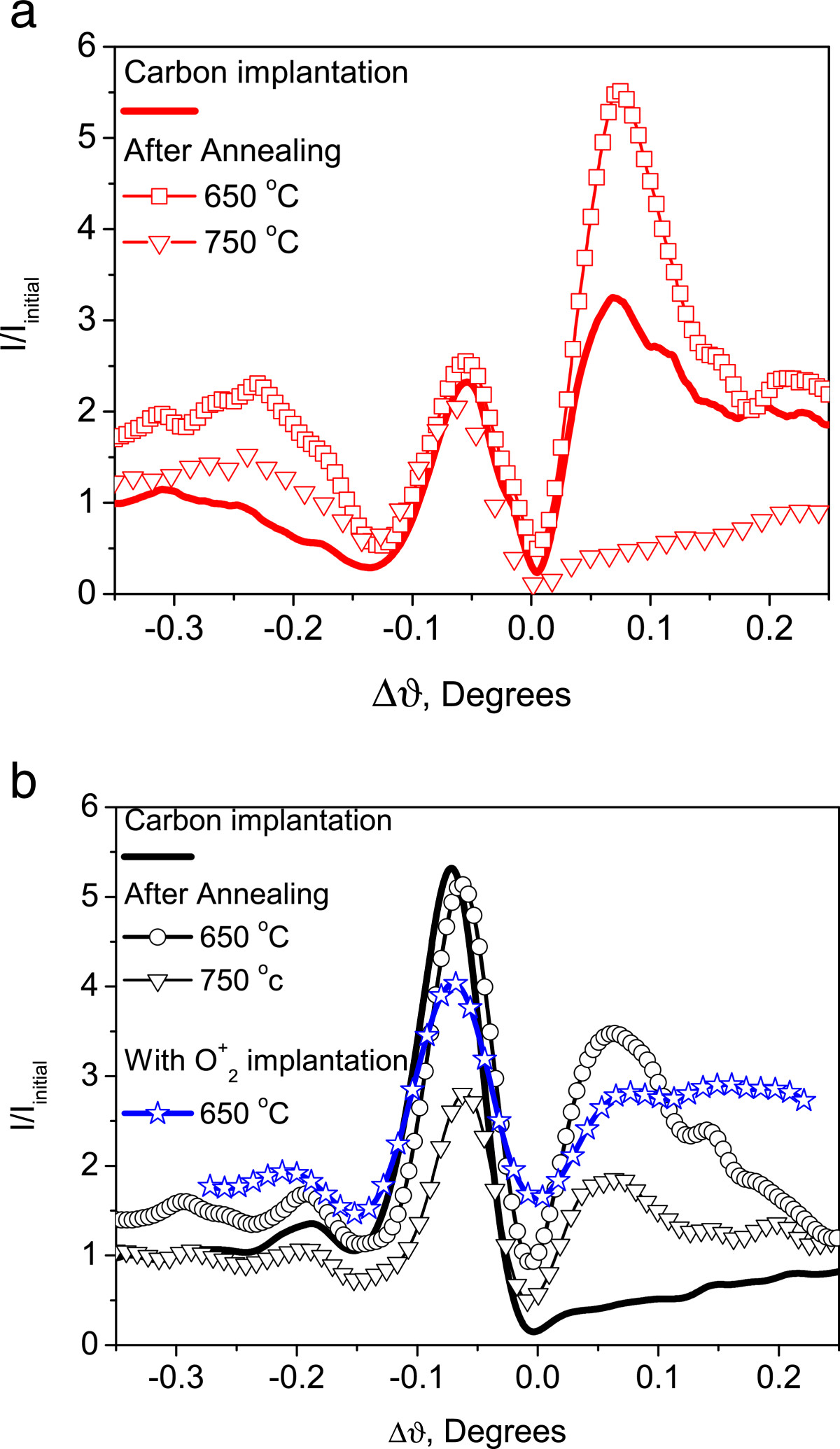
**Distributions of the DXS signal intensity for Cz-Si (a) and FZ-Si (b).**

In the DXS measurements, the distribution of the X-ray scattered intensity (normalized to that of the referential un-implanted crystal) in the direction parallel to the reciprocal lattice vector *q*_z_ (*q*_z_ ~ Δϑ) is presented in Figure 4.

It follows from the analysis of the DXS curves in Figure 4a,b that the sample contains both vacancy-type clusters, which are responsible for the peaks at Δϑ < 0, and interstitial defects, which are responsible for the signal at Δϑ > 0[11]. For the Cz-Si case, an increase in scattering intensity for the Δϑ > 0 and a small decrease of the signal for Δϑ < 0 are observed when the annealing temperature increases from 560°C to 650°C. At *T* = 750°C, there is a substantial intensification of scattering within the region of Δϑ > 0. For the FZ-Si case, a maximum is observed in the scattering region governed by the vacancy-type defects, and for Δϑ > 0, its intensity is less than for the Cz-Si sample.

The increased scattering at Δϑ > 0 for the FZ-Si + implanted oxygen samples is associated with the oxygen precipitation due to the interstitial nature of SiO_*x*_ clusters. It is also accompanied by the decrease in scattering by vacancy-type defects in these samples, Figure 4b. Since the profiles of carbon and oxygen impurity distribution are coincident for this case, we conclude about the vacancy-type defect compensation by the precipitation of oxygen.

It should be noted that annealing at *T* = 650°C of the FZ-Si samples results in higher scattering intensity for Δϑ > 0 but almost does not affect the scattering for the Δϑ < 0 region. This is an indication that the regions of vacancy and interstitial defect types are located at different depths. In the case of additional oxygen implantation, there is no such spatial separation, since oxygen is distributed over a 300-nm depth range, overlapping both defect-type regions.

**Figure 5:**
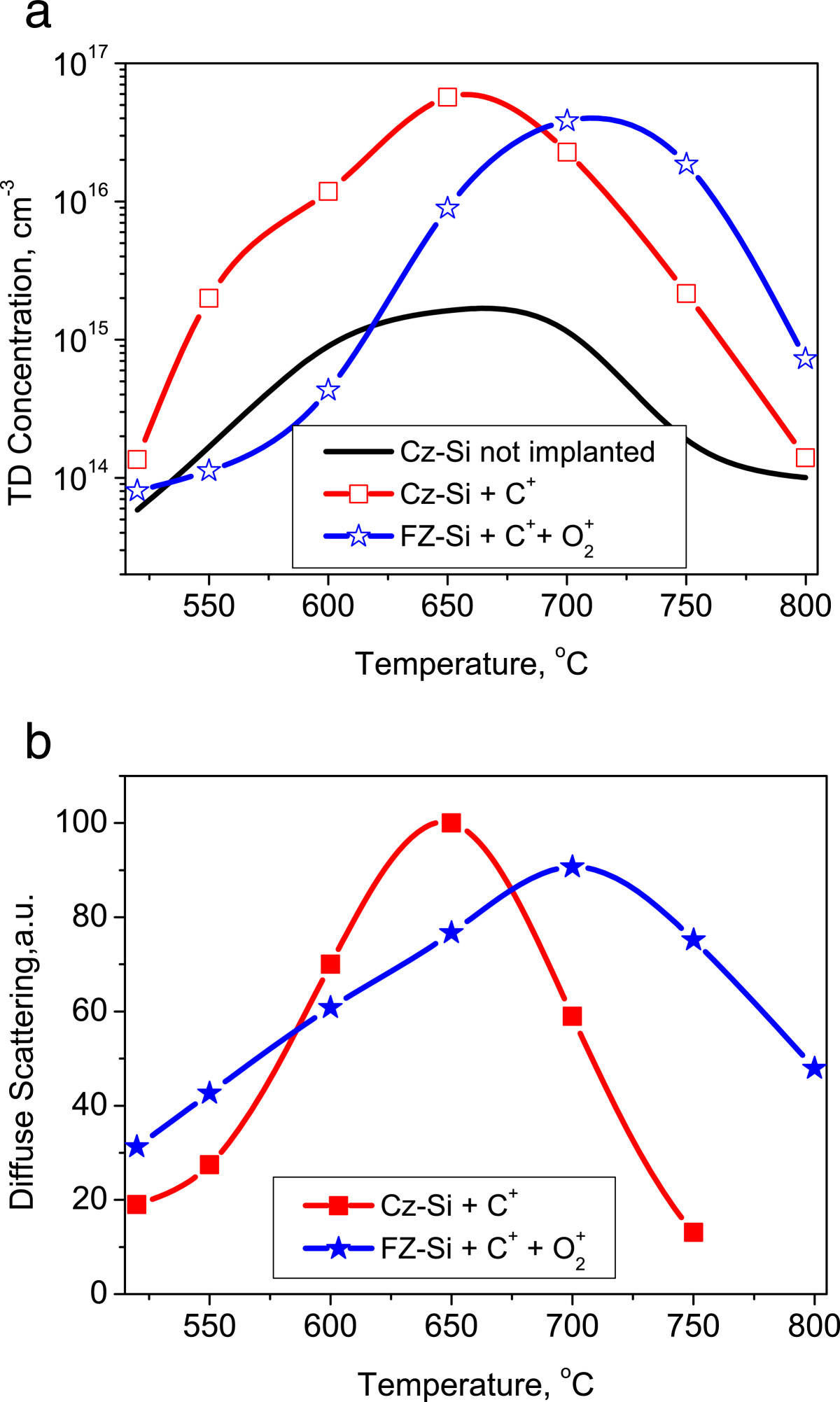
**TD concentration and DXS intensity vs annealing temperature in the region of interstitial defects.** Dependencies of the TD concentration on annealing temperature **(a)** in the as-grown Cz-Si volume, in the carbon-implanted Cz-Si surface layer, and in high-resistivity FZ-Si implanted with carbon and oxygen ions; dependence of the DXS in the region of interstitial defects on annealing temperature **(b)** for Cz-Si (carbon implanted) and FZ-Si (carbon and oxygen implanted).

The dependencies of the TD center concentration on annealing temperature are shown in Figure 5a. For the *n*-Cz-Si sample (doped with 8 × 10^17^ cm^-3^ carbon during crystal growth), the maximum concentration of TD reaches approximately 2 × 10^15^ cm^-3^ after annealing for over 100 h at 650°C[6]. In the case of carbon-implanted Cz-Si, the effective concentration of thermal donors at this annealing temperature almost reaches 1 × 10^17^ cm^-3^, which is roughly two orders of magnitude higher than in the *n*-Cz-Si sample mentioned above. The maximum TD concentration on both curves is achieved after annealing at 650°C.

For FZ-Si implanted with carbon and oxygen ions, this temperature dependence is similar, but the maximum concentration is somewhat lower than in Cz-Si and is observed at *T* = 700°C. In Figure 5b, we present the temperature dependence of the X-ray scattering intensity in DXS for Δϑ > 0. This intensity is proportional to the interstitial defect volume. One can see that the DXS signal's temperature dependence correlates with that for donor concentration. The experimental data in Figure 5a for the referential Cz-Si sample (without implantation) were obtained by Hall effect measurements. In the case of samples implanted with carbon, the TD concentration was calculated from the conductivity measurements of implanted Si layer after annealing.

## Discussion

The above presented results indicate that annealing the Cz-Si crystals with high carbon content in the temperature range between 520°C and 800°C leads to TDs formation. This effect is not dependent on the method of reaching the high content of C_s_ in Si: the latter can be doped with carbon in the process of crystal growth or by ion implantation. It is known that the SiO_*x*_ cluster formation during annealing provides for the TDs activity. The carbon atom serves as a nucleation site for the growing SiO_*x*_ cluster. Each cluster includes one carbon atom and a few oxygen atoms[6, 12–14]. The TDs concentration is determined by the carbon content in the C_s_ state, the total oxygen content inside the Si crystal, and annealing parameters. The TD ionization energy *E*_*i*_ is ≥12 meV, which is fully consistent with the model of thermal donors-II (new thermal donors) for Si crystals proposed in[12]. In this model, the *E*_*i*_ dependence on the oxygen cluster size was established. According to this model, the small ionization energy is determined by shallow potential wells occurring in the vicinity of the small size oxygen precipitates, which takes place in carbon-enriched Si crystals.

In the FZ-Si, the TD centers of the similar nature also arise after a low-temperature annealing of Si crystals implanted with carbon, see Figures 2a and3a. Since FZ-Si does not contain a sufficient amount of oxygen, the latter penetrates through the screen oxide layer into the silicon surface layer due to recoil atoms during implantation, as is shown in Figure 1b. Therefore, the TD concentration and the thickness of the TD formation layer depend on embedded oxygen amount, the latter in its turn being dependent on the screen oxide thickness. For different oxide thicknesses, not only the amount of recoil atoms, but also the thickness and location of the TD center generation region are changed. Our calculations (Figure 1b) have shown that for the screen oxide thickness of 5 nm, a 20-nm thick region is created where the oxygen concentration is sufficient for effective TD formation. There is also an oxygen supersaturation layer of approximately 6-nm thickness in the vicinity of the Si/SiO_2_ interface. It leads to the generation of large SiO_2_ precipitates without any electrical donor activity. In the case of additional oxygen implantation into the FZ-Si, there is an optimal oxygen dose (approximately 8 × 10^13^ cm^-2^) at which the TD concentration reaches its maximum. Increasing the dose above the optimal value leads to large precipitate growth and elimination of electrical TD activity. The ionization energy of TD dopants in Cz-Si is equal to 0.012 eV; while in FZ-Si + implanted oxygen wafers, it reaches the value of 0.014 eV (Figure 3b). This ionization energy difference originates from the larger size of SiO_*x*_ clusters, which are formed in the wafer with additional implanted oxygen. We were unable to determine the activation energy for the case of FZ-Si without oxygen implantation. In this case, the layer where the TDs are formed is extremely thin (approximately 10 nm), thus rendering *C(V)* measurements at varying temperatures unreliable, due to influence of the Schottky diode contribution.

In FZ-Si crystals, a high intensity of X-ray scattering from the vacancy complexes after low-temperature annealing has been observed. It remains almost unchanged with the varying annealing temperature. This effect is associated with embedding the carbon atoms into the interstitial positions. In the Cz-Si sample, the influence of vacancy defects is compensated by the SiO_*x*_ cluster growth. For the FZ-Si sample, the oxygen precipitation takes place within the region of recoil atom distribution, so the regions of carbon presence and oxygen precipitation are spatially separated.

An additional proof of this fact is the presence of intensity oscillations in the X-ray scattering. It clearly hints on the layered distribution of defects in the investigated structures. We have calculated the period of stratification within the range of 100 to 120 nm. This is the distance that separates the precipitated oxygen and carbon in the substitution positions. The situation is reversed after additional oxygen implantation, e.g. in Figure 4b where the vacancy component compensation is observed in the approximately 300-nm wide region, which includes the carbon-rich region. It is important to point out that large SiO_2_ precipitates are always formed in the carbon-containing region. Their presence leads to deviations of the forward-biased part of the *I(V)* characteristic from the ideal ‘diode type’ shape and certain current instabilities in these structures.

## Conclusions

Our work compares the mechanisms of thermal donor (TD) center formation in silicon grown using the float zone (FZ-Si) and Czochralski (Cz-Si) methods as a result of ion implantation of carbon. The above presented data describe the kinetics of the TD center formation and transformation of the surrounding silicon crystalline structure after annealing. In addition, we have investigated the processes of the TD center formation as a result of both carbon and oxygen implantation into the FZ-Si, revealing an important role of recoil oxygen atoms in the TD center formation. The concentration of these atoms in the Si surface layer can reach approximately 10^18^ cm^-3^ and higher, depending on the implantation dose. This is comparable to typical oxygen concentrations in Cz-Si wafers. The recoil oxygen atoms play the crucial role in the TD center generation, especially in the FZ-Si.

## Authors’ original submitted files for images

Below are the links to the authors’ original submitted files for images. Authors’ original file for figure 1 Authors’ original file for figure 2 Authors’ original file for figure 3 Authors’ original file for figure 4 Authors’ original file for figure 5 Authors’ original file for figure 6 Authors’ original file for figure 7 Authors’ original file for figure 8 Authors’ original file for figure 9 Authors’ original file for figure 10
