# Supplementary figures and images for: Structural and electrical properties of oxygen complexes in Cz and FZ silicon crystals implanted with carbon ions
Source: Nanoscale Res Lett. 2014 Dec 23;9:693. doi: 10.1186/1556-276X-9-693 (PMC4967622; doi:10.1186/1556-276X-9-693)

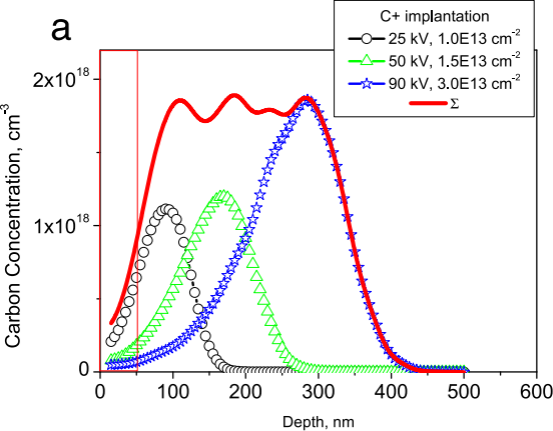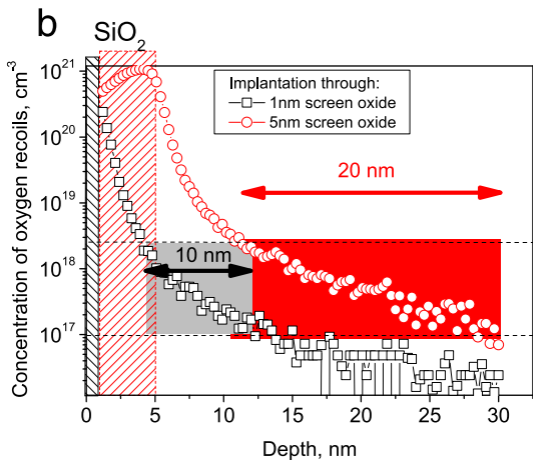

Supplement: Supplementary file 1 — Authors’ original file for figure 1 [file 11671_2014_2411_MOESM1_ESM.pdf]

**a**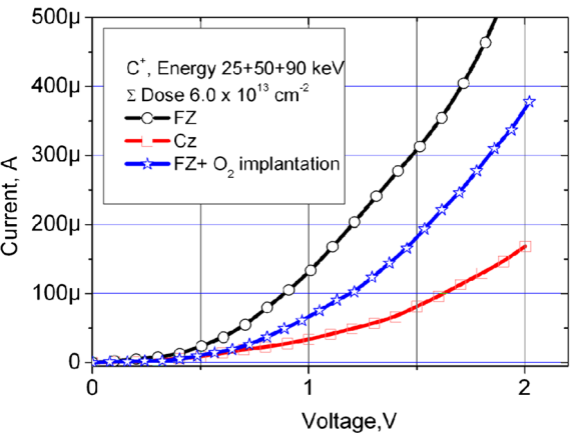**b**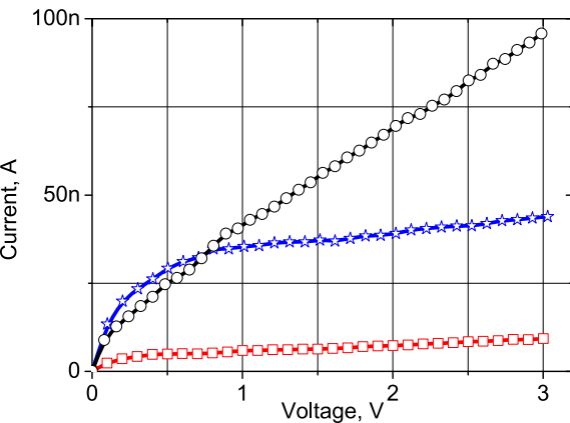

Supplement: Supplementary file 2 — Authors’ original file for figure 2 [file 11671_2014_2411_MOESM2_ESM.pdf]

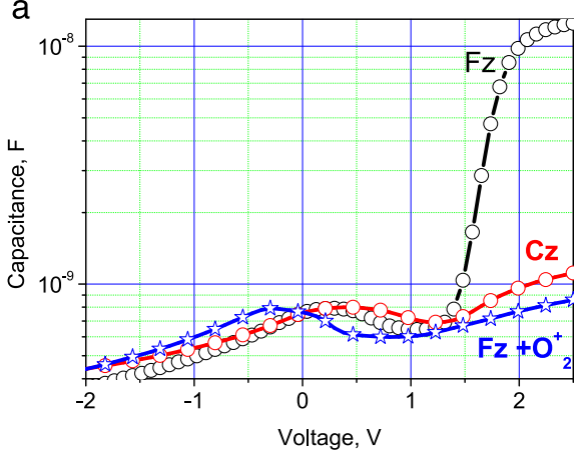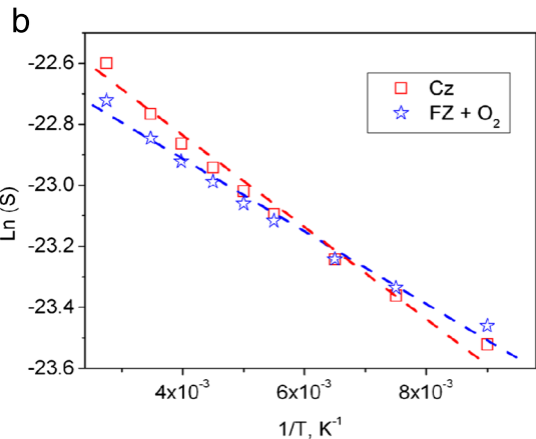

Supplement: Supplementary file 3 — Authors’ original file for figure 3 [file 11671_2014_2411_MOESM3_ESM.pdf]

**a**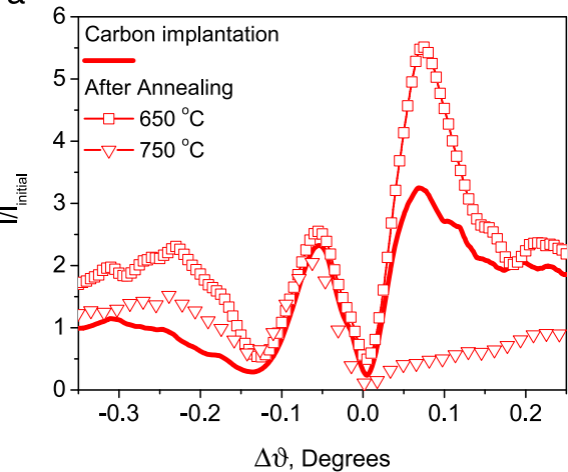**b**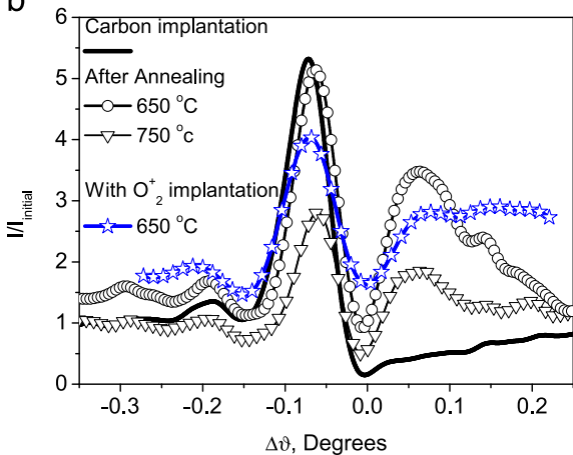

Supplement: Supplementary file 4 — Authors’ original file for figure 4 [file 11671_2014_2411_MOESM4_ESM.pdf]

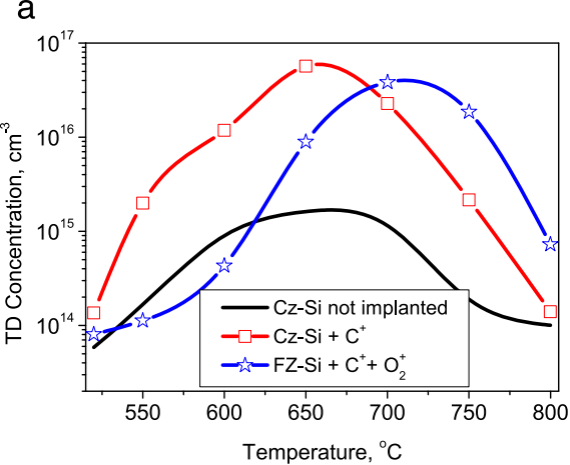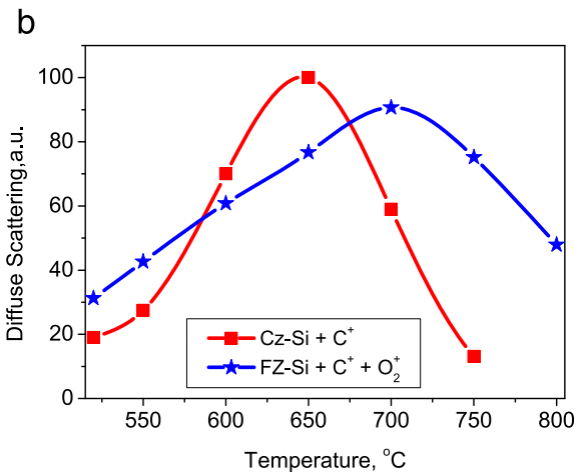

Supplement: Supplementary file 5 — Authors’ original file for figure 5 [file 11671_2014_2411_MOESM5_ESM.pdf]

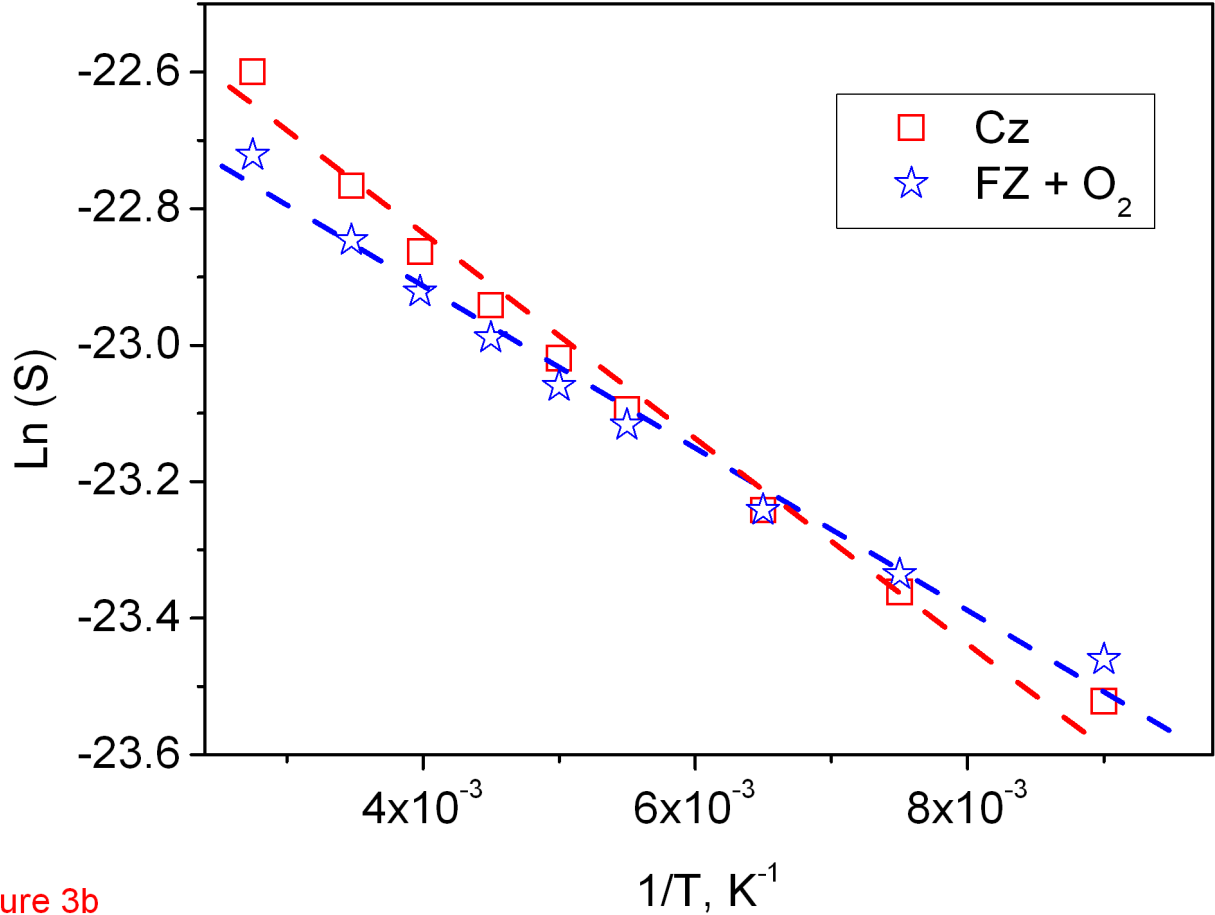

Figure 3b

Supplement: Supplementary file 6 — Authors’ original file for figure 6 [file 11671_2014_2411_MOESM6_ESM.pdf]

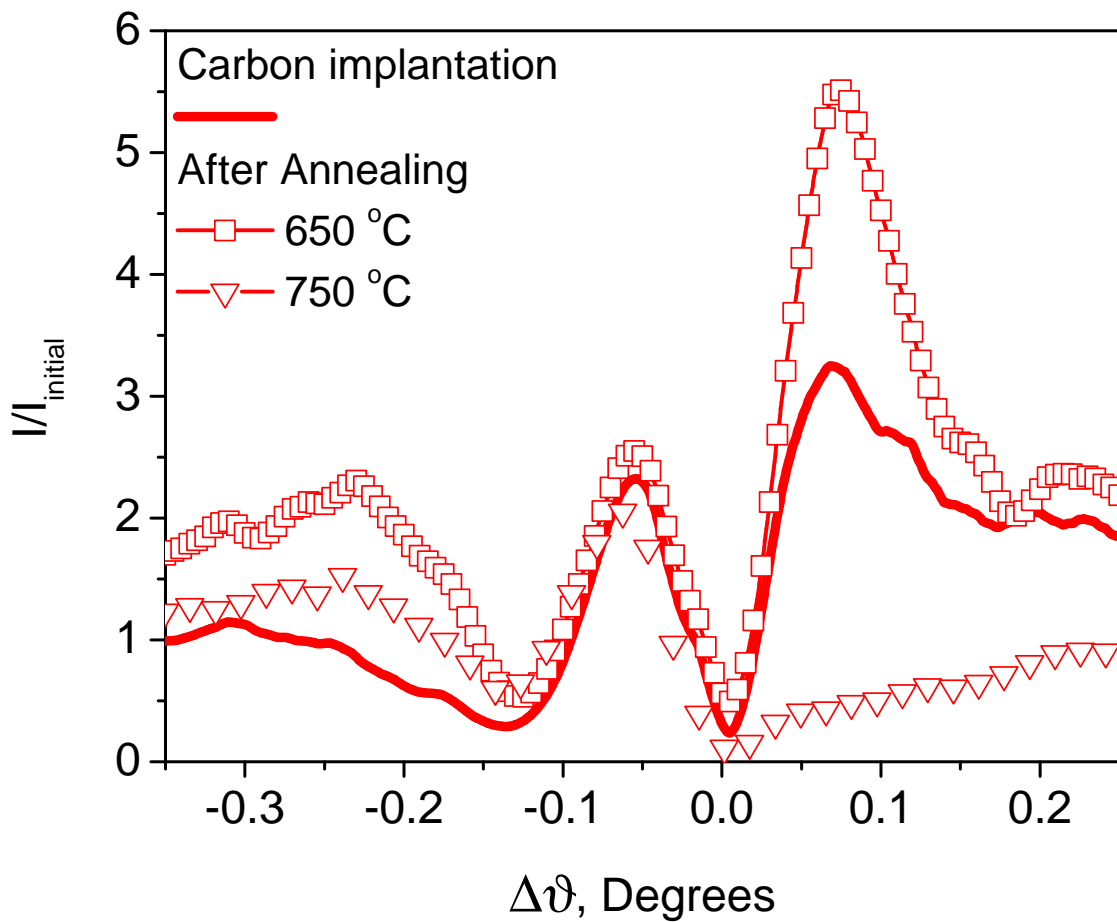

Supplement: Supplementary file 7 — Authors’ original file for figure 7 [file 11671_2014_2411_MOESM7_ESM.pdf]

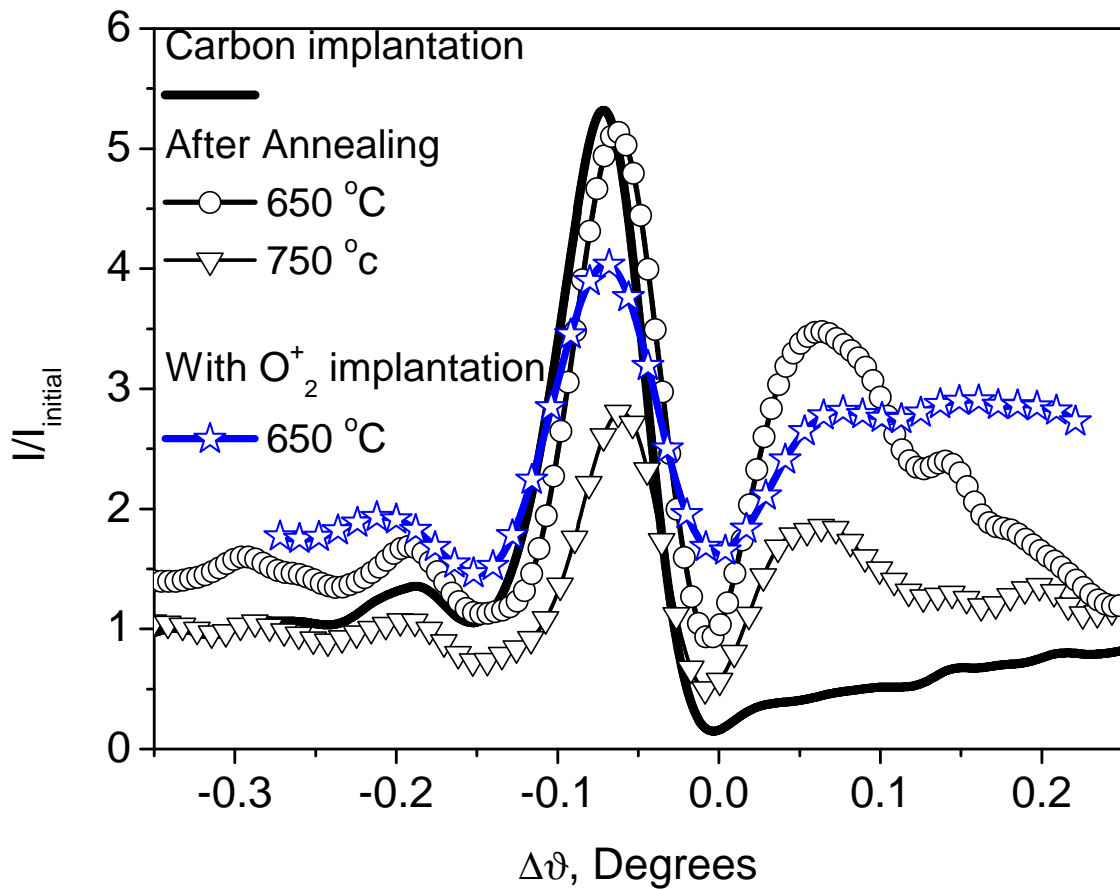

Supplement: Supplementary file 8 — Authors’ original file for figure 8 [file 11671_2014_2411_MOESM8_ESM.pdf]

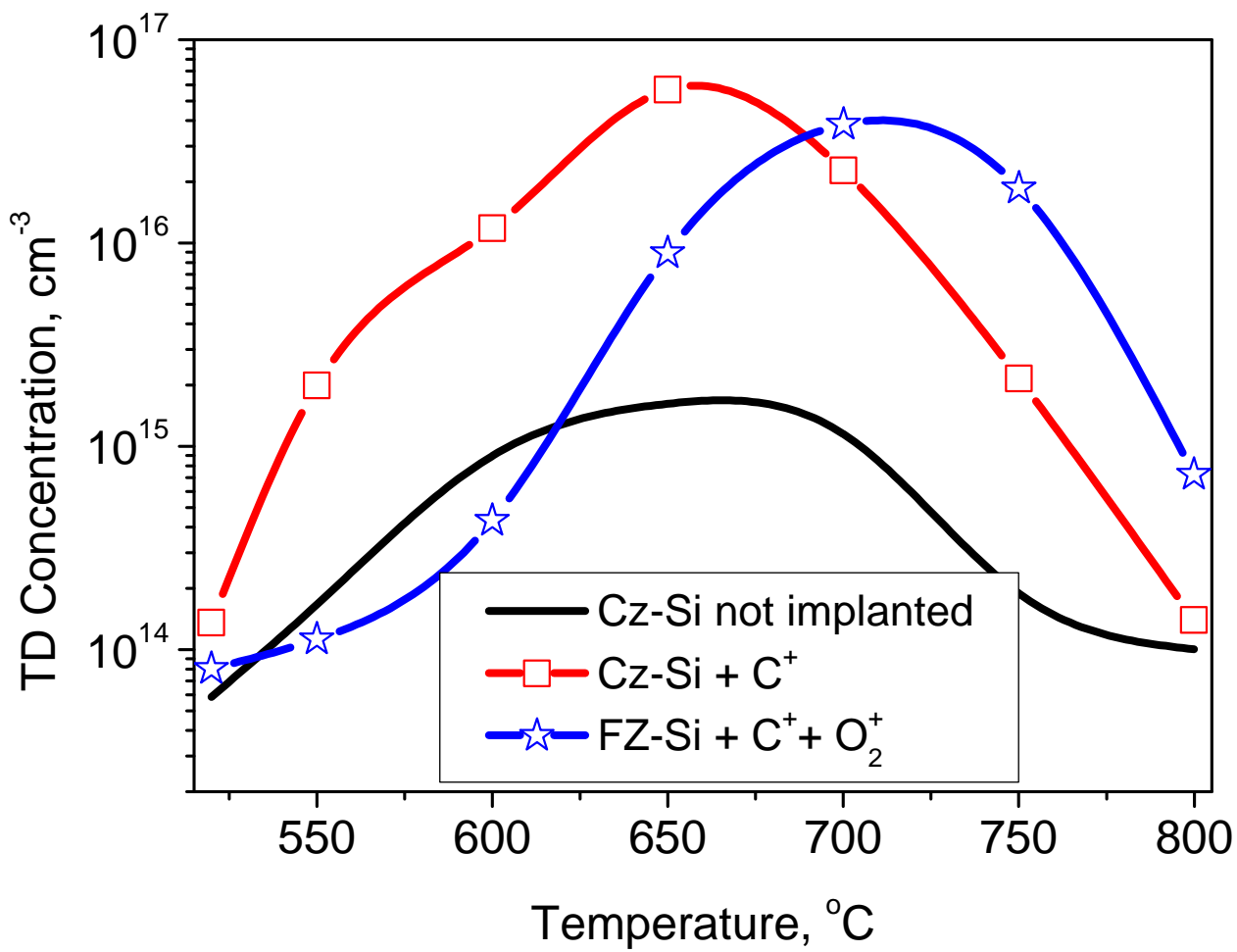

Supplement: Supplementary file 9 — Authors’ original file for figure 9 [file 11671_2014_2411_MOESM9_ESM.pdf]

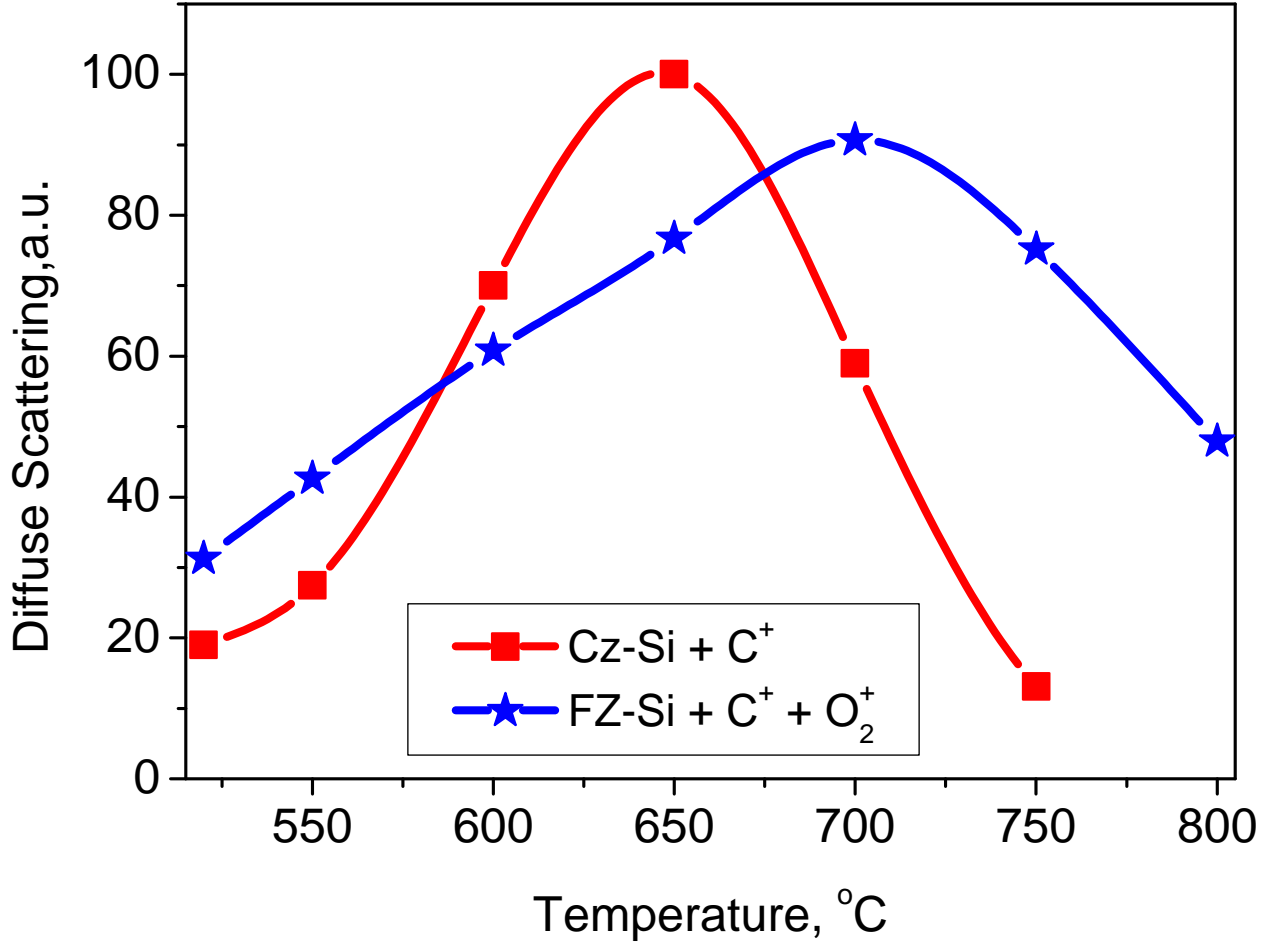

Supplement: Supplementary file 10 — Authors’ original file for figure 10 [file 11671_2014_2411_MOESM10_ESM.pdf]
